# Common cell lysis procedures distort ribosome profiling analyses of gene expression

**DOI:** 10.1186/s13059-025-03651-1

**Published:** 2025-08-11

**Authors:** Aoife O’Connell, Alla D. Fedorova, Patrick B. F. O’Connor, Alexander V. Zhdanov, Pavel V. Baranov, Gary Loughran, Dmitry E. Andreev

**Affiliations:** 1 School of Biochemistry and Cell Biology, University College Cork, Cork, Ireland; 2 EIRNA Bio Ltd, Bioinnovation Hub, Food Science & Technology Building, College Road, Cork, Ireland; 3 iEd Hub and School of Pharmacy, University College Cork, Cork, Ireland; 4 Shemyakin-Ovchinnikov Institute of Bioorganic Chemistry, RAS, Moscow, Russia; 5 Belozersky Institute of Physico-Chemical Biology, Lomonosov Moscow State University, Moscow, Russia

**Keywords:** Ribosome profiling, Polysome profiling, Gene expression, Riboseq, Cytoskeleton, Insoluble fraction, Lysis, Nascent peptide

## Abstract

Ribosome profiling is a powerful technique used to study gene expression on a transcriptome-wide scale. It involves sequencing of mRNA fragments protected by ribosomes from ribonuclease digestion. The initial steps commonly involve cell lysis followed by centrifugation and ribonuclease digestion. We find that centrifugation depletes 329 translated mRNAs in HEK293T cells. Many of these mRNAs encode cytoskeleton proteins. This suggests that the expression of a subset of mRNAs may be significantly underestimated in most ribosome profiling experiments. We show that omitting the centrifugation step after cell lysis can resolve this issue.

**Supplementary Information:**

The online version contains supplementary material available at 10.1186/s13059-025-03651-1.

## Background

Each translating ribosome protects ~ 30 nucleotides of mRNA from ribonuclease digestion [1]. The ribosome profiling (riboseq) technique, developed in 2009 [2], is based on the deep sequencing of ribosome-protected mRNA fragments generated by ribonuclease digestion. Parallel sequencing of undigested mRNA allows a quantitative measure of ribosome density relative to mRNA levels to determine translation efficiency. Variants of the riboseq protocol have been developed that allow for low input/single-cell analysis of ribosome occupancy [3–6], di-some profiling of collided ribosomes [7, 8], and profiling of scanning ribosome complexes [9].

All riboseq protocols begin with cell lysis followed by the removal of cellular debris and insoluble material by fast centrifugation (clarification) before proceeding to ribonuclease digestion. Lysis buffer composition should ideally ensure quantitative mRNA extraction while maintaining mRNA-bound ribosomes while allowing for efficient ribonuclease activity. For riboseq in mammalian cells, lysis is typically achieved using non-ionic detergents, and 1% Triton X100 is used in 45 out of 68 recent riboseq studies (Additional file 1: Table S1). Whether these lysis conditions allow extraction of all ribosome-bound mRNAs has not been thoroughly explored. We present evidence that common sample preparation protocols deplete a specific fraction of translated mRNAs, likely those associated with cytoskeletal components.

## Results and discussion

**Fig. 1:**
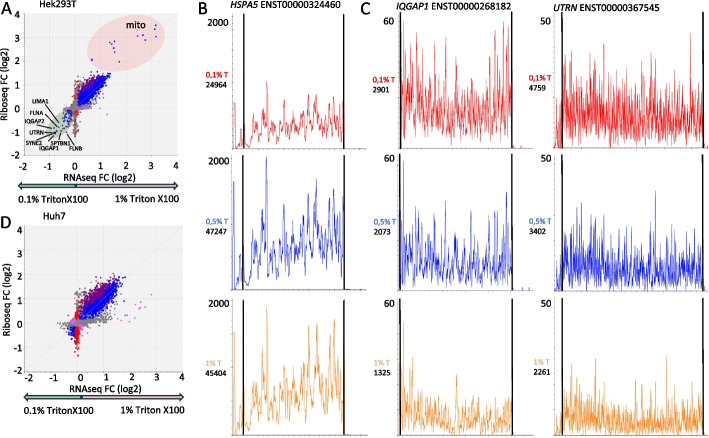
Riboseq lysis buffers with high Triton-X100 concentrations deplete a subset of translated mRNAs. **A** Comparison plot (DEseq2) showing riboseq and RNAseq analyses of HEK293T cells with the ST (1.0% Triton X100) and LT (0.1% Triton X100) lysis buffers. Results are color coded as follows: blue—transcriptional regulation (no change in translation efficiency), red—regulation exclusively by translation, pink—regulation exclusively by transcription without corresponding changes in riboseq (buffering), violet—translational regulation with the transcriptional regulation change in same direction. Names are indicated for selected differentially expressed genes enriched in the LT lysates. **B** Riboseq profile of the ER-localized *HSPA5* mRNAs enriched in the ST lysates. Coding sequence boundaries are indicated by black lines. Red, blue and orange plots correspond to 0.1%, 0.5%, and 1% Triton X100, respectively. The number of riboseq reads aligned to individual mRNA isoform under each lysis condition are shown on the left. **C** Riboseq profiles of *IQGAP1* and *UTRN* mRNAs, both depleted in the ST lysates. **D** Comparison plot (DEseq2) showing riboseq and RNAseq analyses of Huh7 cells using the ST and LT lysis buffers. For **B** and **C**, read counts were normalized by the factor differences between library sizes

Using HEK293T cells cultured under standard conditions, we compared riboseq and RNAseq generated with lysis buffers containing three different concentrations of Triton X100 (0.1%, 0.5%, and 1%). First, we compared the low Triton X100 lysis buffer (LT − 0.1%) with the standard Triton X100 buffer (ST − 1.0%) (Fig. 1A, Additional file 2: Table S2) [10]. Lysates obtained with the ST buffer are enriched in mitochondrial and ER-bound mRNA fragments compared to those obtained with LT buffer in both riboseq and RNAseq experiments (Fig. 1B). This indicates that the standard 1% Triton X100 lysis conditions effectively disrupt the mitochondrial and ER membranes. However, we also observed increased expression of 68 cytosolic mRNAs when using the LT buffer compared to the ST buffer (Additional file 2: Table S2). The concurrent increase in riboseq and RNAseq fragments suggests that these mRNAs are translated and not translationally silenced (where only an increase in RNAseq would be observed). The most abundant mRNAs enriched in the LT buffer are *FLNA*,* FLNB*,* IQGAP1*,* IQGAP2*,* SYNE2*,* SPTBN1*,* UTRN*, and *LIMA1* (Additional file 2: Table S2)*.* All these transcripts encode proteins involved in cytoskeletal organization. To rule out the possibility that their differential expression could be attributed to other technical artifacts, such as overamplification of sparse mRNA fragments leading to spiky read distributions, we analyzed the riboseq signals for their individual mRNA isoforms (Fig. 1C). We observed uniform riboseq read distributions along the coding sequences, which progressively decreased as Triton X100 concentrations increased to 0.5% and 1%. Similar experiments with the Huh7 cell line did not show the same enrichment in LT buffer (Fig. 1D, Additional file 3: Table S3). This may be explained by differences in cytoskeletal organization between Huh7 and HEK293T cells. Taken together, these data suggest that the standard 1.0% Triton X100 lysis buffer may not extract all mRNAs.

**Fig. 2:**
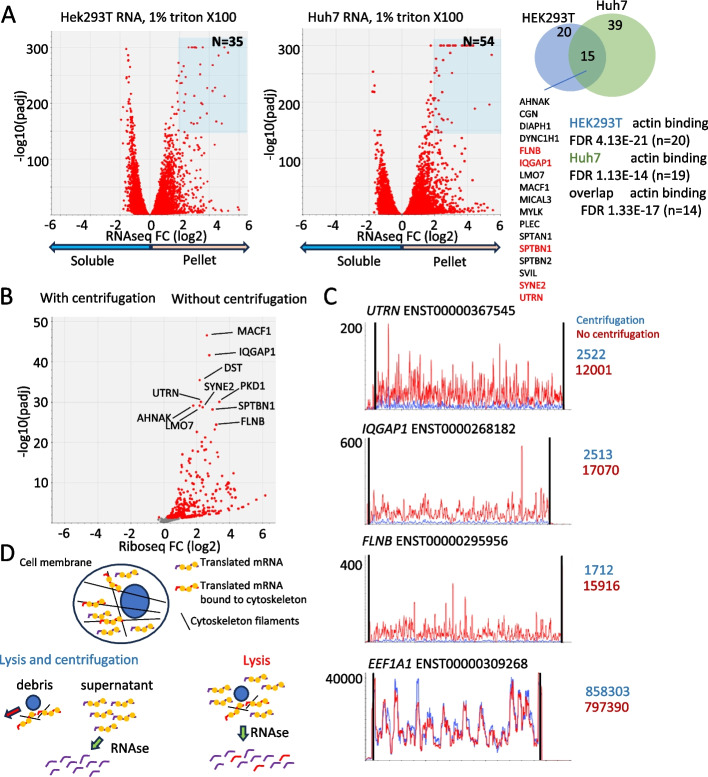
Depleted mRNAs can be recovered by omission of the lysate clarification step. **A** RNAseq, differential expression (DEseq2) of RNA in the soluble fraction vs RNA isolated from the cell debris pellet. mRNAs that are enriched in the pellet fraction are highlighted with blue boxes. Gene Ontology (GO) analysis is summarized on the right. List of mRNAs enriched in pellets of both HEK293T and Huh7 cells is provided below the Venn diagram. Genes which were enriched in riboseq experiments using the LT buffer are marked in red. **B** Riboseq analysis of differential gene expression in HEK293T cells using standard lysis protocols with (left side) and without centrifugation (right side). **C** Riboseq profiles of selected “cytoskeletal” mRNAs, *UTRN*, *IQGAP1*, and *FLNB*. *EEF1A1* riboseq profile is shown as a control. Riboseq reads aligned to mRNA for each condition are shown as colored numbers on the right side of the plots. Read counts were normalized by the factor differences between library sizes. **D** Model describing mRNA localization to the cytoskeleton and its depletion during the centrifugation step

Because mRNAs encoding cytoskeleton proteins are depleted from the standard 1% Triton X100 lysates, we hypothesized that they may be pelleted together with the cell debris during centrifugation. To test this idea, we extracted RNA from both the supernatant (soluble fraction) and the pellet (insoluble fraction), and then performed RNAseq. For differential gene expression analysis, we used DESeq2 [11]. A number of mRNA species were enriched in the insoluble fraction for HEK293T (4474 mRNAs, Additional file 4: Table S4) and Huh7 (4445 mRNAs, Additional file 5: Table S5) (Fig. 2A). We decided to analyze mRNAs most significantly enriched in the insoluble fraction (fold change > 4, padj < 1e^−150^). Gene ontology analysis [12, 13] shows that many of these mRNAs encode actin-binding proteins (Fig. 2B). Notably, most of the 68 mRNAs enriched in the LT lysates (Fig. 1A) were also enriched in the insoluble fraction of both HEK293T cells (58/4474, fold enrichment 3.3, Mann–Whitney *U* rank test *P*-value = 1.65E − 21) and Huh7 cells (46/4445, fold enrichment 2.2, Mann–Whitney *U* rank test *P*-value = 1.66E − 10).

The pelleting of “cytoskeletal” mRNAs may be explained by their co-translational engagement with the cytoskeleton proteins either through nascent chains or through interactions with RNA binding proteins that interact with the cytoskeleton [14–18]. To test this hypothesis, we compared ribosome profiling data generated using HEK293T cell lysates, which were produced with or without the centrifugation step. Omission of the centrifugation step had no significant impact on triplet periodicity, read length distribution, and metagene profiles, which were of good quality under both conditions (Additional file 6: Fig. S1). However, it slightly reduced the proportion of reads that mapped to mRNAs (72% with centrifugation vs. 64% without) and slightly increased the ratio of RPFs that mapped to certain non-coding RNAs (5% vs. 7%, Additional file 7: Fig. S2). We found that among 330 differentially expressed mRNAs (Fig. 2B and C), 329 mRNAs were enriched in non-clarified samples (Fig. 2D, right side). One mRNA was enriched in the clarified sample, although this effect was rather marginal (*TUBAL3*, log2 fold change − 0,29, Padj 0.036, Additional file 8: Table S6). Supporting our hypothesis, GO analysis of the 329 mRNAs showed significant enrichment of genes involved in actin binding (fold enrichment 5.67, FDR 2.76E − 15), actin filament binding (fold enrichment 7.78, FDR 1.41E − 12), and cytoskeletal protein binding (fold enrichment 3.40, FDR 3.90E − 12). For *MACF1*, we found that two isoforms, long and short, are expressed in HEK293T and Huh7 cells. However, the long mRNA is depleted much more efficiently by centrifugation than the short one (Additional file 9: Fig. S3). This “centrifugation” artifact can therefore impact isoform quantification.

## Conclusions

More accurate quantification of protein synthesis can be achieved by avoiding clarification of cell lysates by centrifugation. Alternative approaches for recovering RNA derived from mRNAs encoding cytoskeletal proteins may be possible. This consideration applies not only to ribosome profiling but also to other related techniques such as polysome sequencing (aka RNC-seq).

## Methods

### Ribosome profiling

Ribosome profiling was adapted from McGlincy, Ingolia and colleagues [19, 20] with modifications to the procedure. HEK293T (ATCC) and Huh7 (Thermo Fisher) cells were regularly checked for mycoplasma contamination. The absence of mycoplasma was further verified by sequencing analysis of the obtained riboseq and RNAseq data. The cells have not been authenticated. The cells were maintained in DMEM (Sigma-Aldrich) supplemented with 10% FBS (Sigma-Aldrich), 1 mM L-glutamine (Sigma-Aldrich) and penicillin–streptomycin (Sigma-Aldrich), and cultured at 37 °C and 5% CO_2_. Once cells had reached ~ 70% confluency, the media was aspirated and the cells were washed with ice-cold 1X PBS with 0.1 mg/ml cycloheximide (CHX). The cells were lysed with ice-cold polysome lysis buffer (PLB) (20 mM Tris–Cl pH 7.5, 150 mM NaCl, 5 mM MgCl2, in nuclease-free water (NFW), supplemented with 1 mM DTT, 0.1 mg/ml CHX, 25U/ml Turbo DNase (Invitrogen), and either 1%, 0.5%, or 0.1% Triton X100 (Sigma-Aldrich)) in triplicate. Triton X100 was diluted in un-supplemented PLB before use. Cell lysates were incubated on ice for 10 min then centrifuged at 10,000 × *g* for 5 min at 4 °C to pellet cell debris. RNA concentration of the supernatant was measured using the Qubit 4.0 Fluorometer and Qubit RNA Broad Range (BR) Assay Kit (Invitrogen). Aliquots were taken for riboseq (15 µg) and RNAseq (5 µg) library preparation. For samples without post-lysis cell debris pelleting, one quarter of the lysate was centrifuged at 10,000 × *g* for 5 min at 4 °C to pellet cell debris and discard prior to aliquoting and digestion. Another one quarter was reserved for RNAseq, while the remaining half of the lysate was aliquoted and RNase I digestion was proceeded on the crude lysate.

For RNAseq, total RNA was extracted from both lysate and pelleted cell debris for each Triton X100 condition using TRIzol Reagent (Invitrogen) followed by clean-up using the RNA Clean and Concentrator-5 Kit (Zymo Research), both according to manufacturers’ instructions. The RNA concentrations and RNA Integrity Number were measured using the 2100 Bioanalyzer System and the RNA 6000 Nano Assay Kit (Agilent). The RNAseq libraries were then generated with rRNA depletion at GENEWIZ (Azenta Life Sciences, Germany).

For riboseq, lysates were treated with 35U RNase I (10 U/µl, Biosearch Technologies) at 23 °C for 1 h at 400 rpm. For all samples, 10 µl 10% SDS was then added and the RPFs were then purified using the RNA Clean and Concentrator-5 Kit according to manufacturer’s instructions. The RNA was dephosphorylated by treatment with 10U T4 PNK enzyme (New England BioLabs) in a 10-μl reaction supplemented with 10X PNK buffer and 20U of SUPERaseIn RNase Inhibitor (20 U/μL, Invitrogen) for 1 h at 37 °C, and then purified using the Oligo Clean and Concentrator Kit (Zymo Research) according to manufacturer’s instructions. RNA was resolved by 15% denaturing polyacrylamide TBE-urea PAGE (containing 1X TBE, 8 M urea, 40% acrylamide (19):bis-acrylamide). Bands corresponding to RNA fragments of 28–30 nt were excised and extracted using the ZR small-RNA PAGE Recovery Kit (Zymo Research) according to manufacturer’s instructions. Contaminating rRNA sequences in the samples were targeted with a custom pool of 20 × 5′-biotinylated subtraction oligos (Table 1). 1.25 µM oligo pool and 20U SUPERaseIn RNase Inhibitor were added to the sample and hybridised at 80 °C for 2 min followed by cooling to 25 °C at 0.1 °C/s. The 20 µl hybridised sample was added to 30 µl pre-washed Dynabeads MyOne Streptavidin C1 (Invitrogen) according to manufacturer’s instructions, and then incubated in a thermomixer at 25 °C for 15 min at 500 rpm. The samples were placed in a magnetic stand and the approx. 50 µl rRNA-depleted-supernatant was removed and purified using the Oligo Clean and Concentrator Kit according to manufacturer’s instructions.

**Table 1:** DNA sequences of biotinylated oligos for human rRNA depletion

Ref	rRNA	Start	End	Sequence (5′ → 3′)
Hu_1	5.8S	86	118	/5Biosg/GGGGCCGCAAGTGCGTTCGAAGTGTCGATGATC
Hu_2	5.8S	132	156	/5Biosg/AAGCGACGCTCAGACAGGCGTAGCC
Hu_3	18S	205	233	/5Biosg/GTTTTGATCTGATAAATGCACGCATCCCC
Hu_4	18S	285	314	/5Biosg/ATCGGCCCGAGGTTATCTAGAGTCACCAAA
Hu_5	18S	840	867	/5Biosg/CATTATTCCTAGCTGCGGTATCCAGGCG
Hu_6	28S	1	34	/5Biosg/TTCAGCGGGTCGCCACGTCTGATCTGAGGTCGCG
Hu_7	28S	194	219	/5Biosg/CTGGGCCTCGATCAGAAGGACTTGGG
Hu_8	28S	935	960	/5Biosg/TCCCTCGGCCCCGGGATTCGGCGAGT
Hu_9	28S	1067	1094	/5Biosg/CGCGCCGTGGGAGGGGTGGCCCGGCCCC
Hu_10	28S	1442	1468	/5Biosg/GGCCGGTGGTGCGCCCTCGGCGGACTG
Hu_11	28S	4060	4100	/5Biosg/GCTCGCCCCCCCGCCTCACCGGGTCAGTGAAAAAACGATCA
Hu_12	28S	4742	4782	/5Biosg/GACAGGCGGGGGACCGGCTATCCGAGGCCAACCGAGGCTCC
Hu_13	28S	4964	4990	/5Biosg/GAAGCAGGTCGTCTACGAATGGTTTAG
Hu_14	28S	5035	5064	/5Biosg/CCCTTGTGTCGAGGGCTGACTTTCAATAGA
Hu_15	18S	1719	1743	/5Biosg/CGAGGGCCTCACTAAACCATCCAAT
Hu_16	18S	1788	1813	/5Biosg/TAGATAGTCAAGTTCGACCGTCTTCT
Hu_17	28S	141	165	/5Biosg/TCTTCCGTACGCCACATGTCCCGCG
Hu_18	28S	709	734	/5Biosg/CTTCCCAGCCGTCCCGGAGCCGGTCG
Hu_19	28S	1722	1751	/5Biosg/TCTAATCATTCGCTTTACCGGATAAAACTG
Hu_20	28S	4036	4060	/5Biosg/AGAGTAGTGGTATTTCACCGGCGGC

The 3′ ends of the RNA were then ligated with a pre-adenylated DNA linker with an 8 nt unique molecular identifier (purchased from IDT, 5′-/5Phos/(N:25252525)(N)(N)(N)(N)(N)(N)(N)AGATCGGAAGAGCACACGTCTGA/3ddc/ overnight at 23 °C. The 15.5-µl reaction was supplemented with 1 µM pre-adenylated linker, 17.5% PEG-8000, 1X T4 RNA Ligase Buffer, 100U T4 RNA Ligase 2, truncated K227Q (200U/μl, New England BioLabs). The ligation reactions were separated by 15% denaturing polyacrylamide TBE-urea PAGE as previously described, and the ligation products were excised and extracted using the ZR small-RNA PAGE Recovery Kit. This RNA–DNA hybrid molecule is used as a template for reverse transcription.

cDNA synthesis and PCR of 3′ ligated products was carried out using the SMARTer smRNAseq Kit for Illumina (Takara Bio) with the following modifications to the procedure. In Section A) Polyadenylation, Poly(A) Polymerase and ATP were not added to the 7.25-µl sample. Instead, only RNase Inhibitor and smRNA Mix 1 were added as described in Step 5. Step 8 was omitted, instead proceeding straight to Section B) cDNA Synthesis where 0.1 nM of a custom reverse transcription primer (purchased from IDT, 5′-GTGACTGGAGTTCAGACGTGTGCTC-3′) was added instead of the 3′ smRNA dT Primer provided. The remaining procedures for Reverse Transcription and PCR were carried out according to the manufacturer’s instructions. The PCR product was purified using the Select-a-Size DNA Clean & Concentrator Kit (Zymo Research) using the ≥ 150 bp (70 µl) protocol, according to the manufacturer’s instructions. Libraries were pooled for deep sequencing on the Illumina NovaSeq System at GENEWIZ (Azenta Life Sciences, Germany).

### Computational analysis of ribosome profiling and RNAseq data

Both stranded mRNAseq libraries and riboseq libraries were sequenced 150PE on Illumina’s Nova-seq 6000 platform to depths of 20 million and 100 million raw read pairs per sample, respectively.

For the riboseq, the second mate from the Illumina paired-end sequencing was discarded as it does not contain any useful information. The single-end raw fastq files have the following sequence structure:

- QQQ—rpf sequence—NNNNNNNN – AGATCGGAAGAGCACACGTCTGAA

The first 3nt (Q) are untemplated additions (usually C or G). These are followed by the sequence of interest. The next 8 nucleotides (N) are the UMI. The adapter sequence is AGATCGGAAGAGCACACGTCTGAA. 

The raw riboseq reads were pre-processed as follows:

1. Cutadapt (version 4.4) [21] was used with parameters -u 3 and -a AGATCGGAAGAGCACACGTCTGAA to remove the untemplated addition and linker sequence.
2. Using EIRNA Bio’s proprietary script, the last 8 nts (i.e., UMI) were copied to the header of each fastq read, and the UMIs were subsequently trimmed from the end of the read. In this way, each of the riboseq fastq files has a read sequence with an associated UMI in the header which was used to collapse PCR duplicates. The riboseq reads were collapsed after filtering out reads that align to rRNA, tRNA, and snoRNA. The RNAseq raw fastq files are paired-end. Using Cutadapt (version 4.4), the following adapter sequences were removed from the RNAseq files:
  Read1 = AGATCGGAAGAGCACACGTCTGAACTCCAGTCA
  Read2 = AGATCGGAAGAGCGTCGTGTAGGGAAAGAGTGT

To remove reads of artefactual origin, both the riboseq and RNAseq were first aligned to rRNA, tRNA, and snRNA/snoRNA using bowtie [22] (version 1.3.1 and settings bowtie -v 3). The reads were then aligned to the human transcriptome based on Gencode version 41 annotation [23]. Genes annotated as pseudogenes, processed_transcripts, and incomplete transcripts were filtered from the transcriptome. Reads were then mapped with bowtie (version 1.3.1 and settings -a -m 100 -l 25 -n 2 –norc) using a two-step approach. First, reads were aligned to transcripts annotated as protein coding. Reads which failed to align to this were next aligned to the remaining transcripts annotated as non-coding. This approach reduces the number of reads which are ambiguously mapped, thereby enabling better estimation of gene expression. Up to 3 mismatches are allowed when mapping the riboseq or the RNAseq sequence reads. This is to account for mapping to a reference sequence where there may be differences with respect to the actual sequence of origin.

Transcriptome alignments were uploaded to EIRNA Bio’s proprietary bioinformatic analysis and visualization platform EIRNA Bio Connect (https://eirnabio.com/eirna-bio-connect/). Uniquely mapping reads are assigned a count of 1. Reads that map to > 1 location are weighted according to the number of gene loci to which the read maps.

### Gene ontology analysis

Analysis was performed with Panther knowledgebase (Pantherdb.org) Panther 19.0 release [24] (Gene Ontology Data Archive [25]). The list of DE genes was analyzed against Homo sapiens reference list (all genes) with annotation data set GO Molecular Function Complete. False discovery rates were calculated with Fisher’s exact test.

## Supplementary Information

Additional file 1: Table S1. Lysis buffer compositions used in 68 recent riboseq studies.

Additional file 2: Table S2. Riboseq and RNAseq analyses (DEseq2) of HEK293T cells lysed with the ST (1.0% Triton X100) and LT (0.1% Triton X100) lysis buffers. Source data were used to generate the plot in Fig. 1A.

Additional file 3: Table S3. Riboseq and RNAseq analyses (DEseq2) of Huh7 cells lysed with the ST (1.0% Triton X100) and LT (0.1% Triton X100) lysis buffers. Source data were used to generate the plot in Fig. 1D.

Additional file 4: Table S4. RNAseq analysis, differential expression (DEseq2) of RNA in the soluble fraction vs RNA isolated from the cell debris pellet of HEK293T cells. Source data were used to generate the plot in Fig. 2A (HEK293T).

Additional file 5: Table S5. RNAseq analysis, differential expression (DEseq2) of RNA in the soluble fraction vs RNA isolated from the cell debris pellet of Huh7 cells. Source data were used to generate the plot in Fig. 2A.

Additional file 6: Fig. S1. Effects of omission of centrifugation on riboseq data quality. A) Reads breakdown; B) Triplet periodicity; C) Read length distribution; D) Metagene profiles centered around start and stop codons.

Additional file 7: Fig. S2. Effects of omission of centrifugation on footprints originated from non-coding RNAs. A) Riboseq analysis of differential gene expression (DEseq2) in HEK293T cells using standard lysis protocols with (left side) and without centrifugation (right side), mapped exclusively on non-coding transcripts. B, C and D – examples of genes with increased footprints in samples without centrifugation. Footprints are color coded by corresponding reading frames. Spurious locations of footprints likely indicate their non-ribosomal origin (e.g. from RNP protection).

Additional file 8: Table S6. Riboseq analysis of differential gene expression in HEK293T cells using standard lysis protocols with and without centrifugation. Source data were used to generate the plot in Fig. 2B.

Additional file 9: Fig. S3. Analysis of the effects of omission of centrifugation on riboseq and RNAseq reads aligned to *MACF1* genomic locus A) Genomic alignments of riboseq with centrifugation (blue) and without centrifugation (red), related to Fig 2B, and alignments of RNAseq from cell supernatant (blue) and cell pellet (red), related to Fig. 2B, and alignments of RNAseq from cell supernatantand cell pellet, related to Fig. 2A. Region where read density increases in centrifugated and supernatant samples is highlighted in blue. First exon of shorter mRNA isoform which likely corresponds to NM_001397473.1 contain multiple translated uORFs which are highlighted by violet arrows. B) RNAseq reads aligned to longer mRNA ENST00000361689 of supernatant and pellet samples related to Fig. 2A.

## Data Availability

All sequencing data generated in this study are accessible at the NCBI Gene Expression Omnibus (GEO) under accession number GSE274847.

## References

[1] Steitz JA. Polypeptide chain initiation: nucleotide sequences of the three ribosomal binding sites in bacteriophage R17 RNA. Nature. 1969;224:957–64. 5360547 10.1038/224957a0

[2] Ingolia NT, Ghaemmaghami S, Newman JR, Weissman JS. Genome-wide analysis in vivo of translation with nucleotide resolution using ribosome profiling. Science. 2009;324:218–23. 19213877 10.1126/science.1168978 PMC2746483

[3] Meindl A, Romberger M, Lehmann G, Eichner N, Kleemann L, Wu J, Danner J, Boesl M, Mesitov M, Meister G, et al. A rapid protocol for ribosome profiling of low input samples. Nucleic Acids Res. 2023;51:e68. 37246712 10.1093/nar/gkad459 PMC10359457

[4] Ozadam H, Tonn T, Han CM, Segura A, Hoskins I, Rao S, Ghatpande V, Tran D, Catoe D, Salit M, Cenik C. Single-cell quantification of ribosome occupancy in early mouse development. Nature. 2023;618:1057–64. 37344592 10.1038/s41586-023-06228-9 PMC10307641

[5] Ferguson L, Upton HE, Pimentel SC, Mok A, Lareau LF, Collins K, Ingolia NT. Streamlined and sensitive mono- and di-ribosome profiling in yeast and human cells. Nat Methods. 2023;20:1704–15. 37783882 10.1038/s41592-023-02028-1 PMC11276118

[6] Li Q, Yang H, Stroup EK, Wang H, Ji Z. Low-input RNase footprinting for simultaneous quantification of cytosolic and mitochondrial translation. Genome Res. 2022;32:545–57. 35193938 10.1101/gr.276139.121 PMC8896460

[7] Meydan S, Guydosh NR. Disome and trisome profiling reveal genome-wide targets of ribosome quality control. Mol Cell. 2020;79(588–602):e586. 10.1016/j.molcel.2020.06.010 PMC7484464 32615089

[8] Arpat AB, Liechti A, De Matos M, Dreos R, Janich P, Gatfield D. Transcriptome-wide sites of collided ribosomes reveal principles of translational pausing. Genome Res. 2020;30:985–99. 32703885 10.1101/gr.257741.119 PMC7397865

[9] Archer SK, Shirokikh NE, Beilharz TH, Preiss T. Dynamics of ribosome scanning and recycling revealed by translation complex profiling. Nature. 2016;535:570–4. 27437580 10.1038/nature18647

[10] O’Connell A FA, O’Connor PB, Zhdanov AV, Baranov PV, Loughran G, Andreev DE. GSE274847. Gene Expression Omnibus. Common cell lysis procedures distort ribosome profiling analyses of gene expression. Available from: https://www.ncbi.nlm.nih.gov/geo/query/acc.cgi?acc=GSE274847; https://www.ncbi.nlm.nih.gov/bioproject/PRJNA1148174. 10.1186/s13059-025-03651-1 PMC12341276 40790224

[11] Love MI, Huber W, Anders S. Moderated estimation of fold change and dispersion for RNA-seq data with DESeq2. Genome Biol. 2014;15:550. 25516281 10.1186/s13059-014-0550-8 PMC4302049

[12] Ashburner M, Ball CA, Blake JA, Botstein D, Butler H, Cherry JM, Davis AP, Dolinski K, Dwight SS, Eppig JT, et al. Gene ontology: tool for the unification of biology. The Gene Ontology Consortium. Nat Genet. 2000;25:25–9. 10802651 10.1038/75556 PMC3037419

[13] Gene Ontology C, Aleksander SA, Balhoff J, Carbon S, Cherry JM, Drabkin HJ, Ebert D, Feuermann M, Gaudet P, Harris NL, et al. The Gene Ontology knowledgebase in 2023. Genetics. 2023;224:iyad031. 36866529 10.1093/genetics/iyad031 PMC10158837

[14] Shiber A. Early insights into co-translational assembly of protein complexes. Nat Rev Mol Cell Biol. 2024;25:515. 38609563 10.1038/s41580-024-00728-w

[15] Williams NK, Dichtl B. Co-translational control of protein complex formation: a fundamental pathway of cellular organization? Biochem Soc Trans. 2018;46:197–206. 29432142 10.1042/BST20170451

[16] Chekulaeva M. Mechanistic insights into the basis of widespread RNA localization. Nat Cell Biol. 2024;26:1037–46. 38956277 10.1038/s41556-024-01444-5

[17] Hopfler M, Hegde RS. Control of mRNA fate by its encoded nascent polypeptide. Mol Cell. 2023;83:2840–55. 37595554 10.1016/j.molcel.2023.07.014 PMC10501990

[18] Das S, Vera M, Gandin V, Singer RH, Tutucci E. Intracellular mRNA transport and localized translation. Nat Rev Mol Cell Biol. 2021;22:483–504. 33837370 10.1038/s41580-021-00356-8 PMC9346928

[19] McGlincy NJ, Ingolia NT. Transcriptome-wide measurement of translation by ribosome profiling. Methods. 2017;126:112–29. 28579404 10.1016/j.ymeth.2017.05.028 PMC5582988

[20] Ingolia NT, Brar GA, Rouskin S, McGeachy AM, Weissman JS. The ribosome profiling strategy for monitoring translation in vivo by deep sequencing of ribosome-protected mRNA fragments. Nat Protoc. 2012;7:1534–50. 22836135 10.1038/nprot.2012.086 PMC3535016

[21] Martin M. Cutadapt removes adapter sequences from high-throughput sequencing reads. EMBnet J. 2011;17:3.

[22] Langmead B, Trapnell C, Pop M, Salzberg SL. Ultrafast and memory-efficient alignment of short DNA sequences to the human genome. Genome Biol. 2009;10:R25. 19261174 10.1186/gb-2009-10-3-r25 PMC2690996

[23] Frankish A, Carbonell-Sala S, Diekhans M, Jungreis I, Loveland JE, Mudge JM, Sisu C, Wright JC, Arnan C, Barnes I, et al. GENCODE: reference annotation for the human and mouse genomes in 2023. Nucleic Acids Res. 2023;51:D942–9. 36420896 10.1093/nar/gkac1071 PMC9825462

[24] Thomas PD, Ebert D, Muruganujan A, Mushayahama T, Albou LP, Mi H. PANTHER: making genome-scale phylogenetics accessible to all. Protein Sci. 2022;31:8–22. 34717010 10.1002/pro.4218 PMC8740835

[25] Carbon S, Mungall C. Gene ontology data archive. Zenodo. 2024. 10.5281/zenodo.10536401.

