# Supplementary material for: Common cell lysis procedures distort ribosome profiling analyses of gene expression
Source: Genome Biol. 2025 Aug 11;26:241. doi: 10.1186/s13059-025-03651-1 (PMC12341276; doi:10.1186/s13059-025-03651-1)
Supplement: Supplementary file 6 — Additional file 6: Fig. S1. Effects of omission of centrifugation on riboseq data quality. A) Reads breakdown; B) Triplet periodicity; C) Read length distribution; D) Metagene profiles centered around start and stop codons. [file 13059_2025_3651_MOESM6_ESM.pptx]

## Slide 1
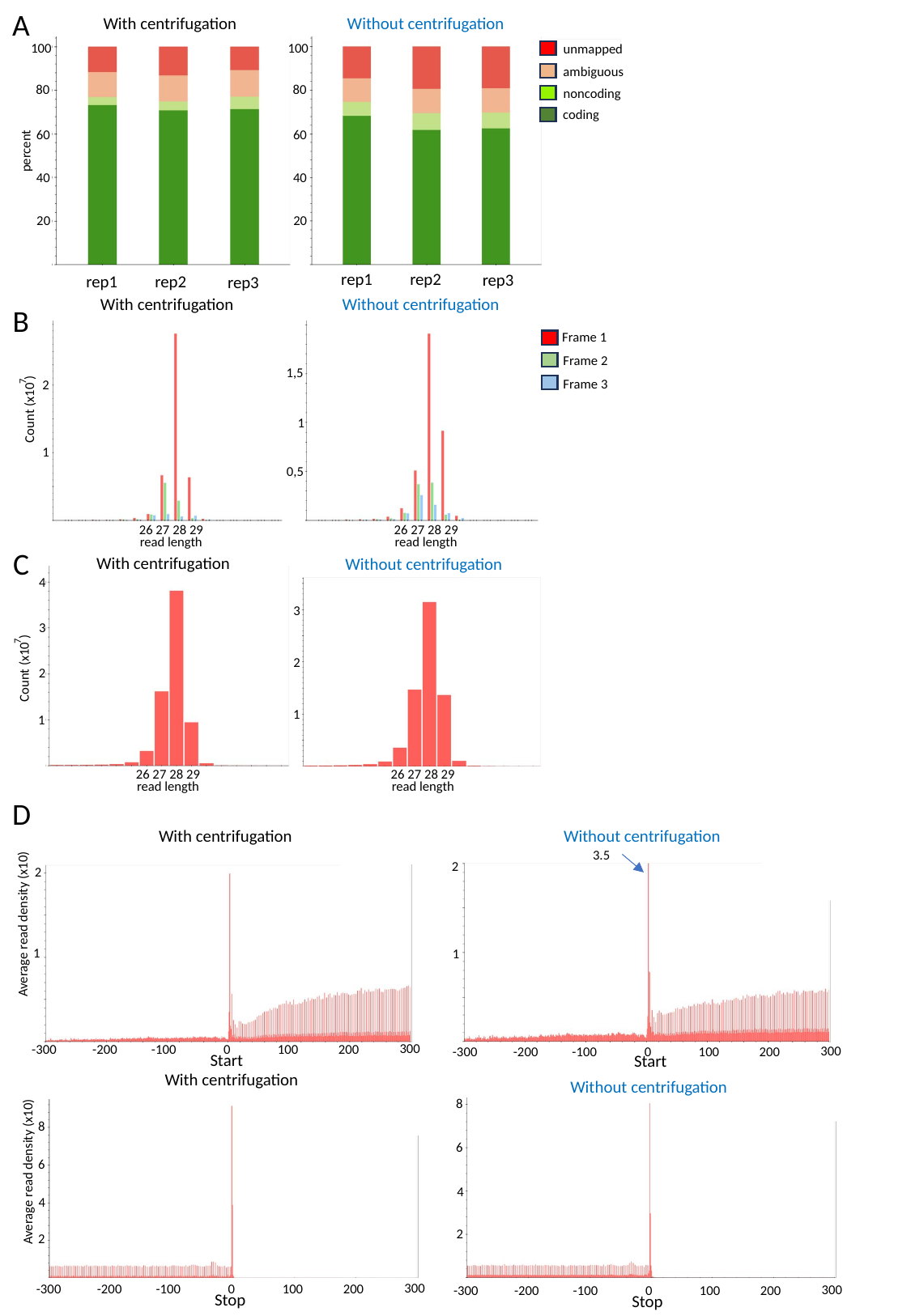

With centrifugation
A
Without centrifugation
100
100
unmapped
ambiguous
noncoding
coding
80
80
60
60
percent
40
40
20
20
rep1
rep2
rep3
rep1
rep2
rep3
With centrifugation
Without centrifugation
B
Frame 1
Frame 2
1,5
Frame 3
2
7
Count (x10 )
1
1
0,5
26 27 28 29
26 27 28 29
read length
read length
C
With centrifugation
Without centrifugation
4
3
3
2
7
Count (x10 )
2
1
1
26 27 28 29
26 27 28 29
read length
read length
D
With centrifugation
Without centrifugation
3.5
2
2
Average read density (x10)
1
1
300
200
-300
-200
0
-100
100
300
200
-300
-200
0
-100
100
Start
Start
With centrifugation
Without centrifugation
8
8
6
6
Average read density (x10)
4
4
2
2
300
200
-300
-200
0
-100
100
300
200
-300
-200
0
-100
100
Stop
Stop
