# Supplementary material for: Common cell lysis procedures distort ribosome profiling analyses of gene expression
Source: Genome Biol. 2025 Aug 11;26:241. doi: 10.1186/s13059-025-03651-1 (PMC12341276; doi:10.1186/s13059-025-03651-1)
Supplement: Supplementary file 7 — Additional file 7: Fig. S2. Effects of omission of centrifugation on footprints originated from non-coding RNAs. A) Riboseq analysis of differential gene expression (DEseq2) in HEK293T cells using standard lysis protocols with (left side) and without centrifugation (right side), mapped exclusively on non-coding transcripts. B, C and D – examples of genes with increased footprints in samples without centrifugation. Footprints are color coded by corresponding reading frames. Spurious locations of footprints likely indicate their non-ribosomal origin (e.g. from RNP protection). [file 13059_2025_3651_MOESM7_ESM.pptx]

## Slide 1
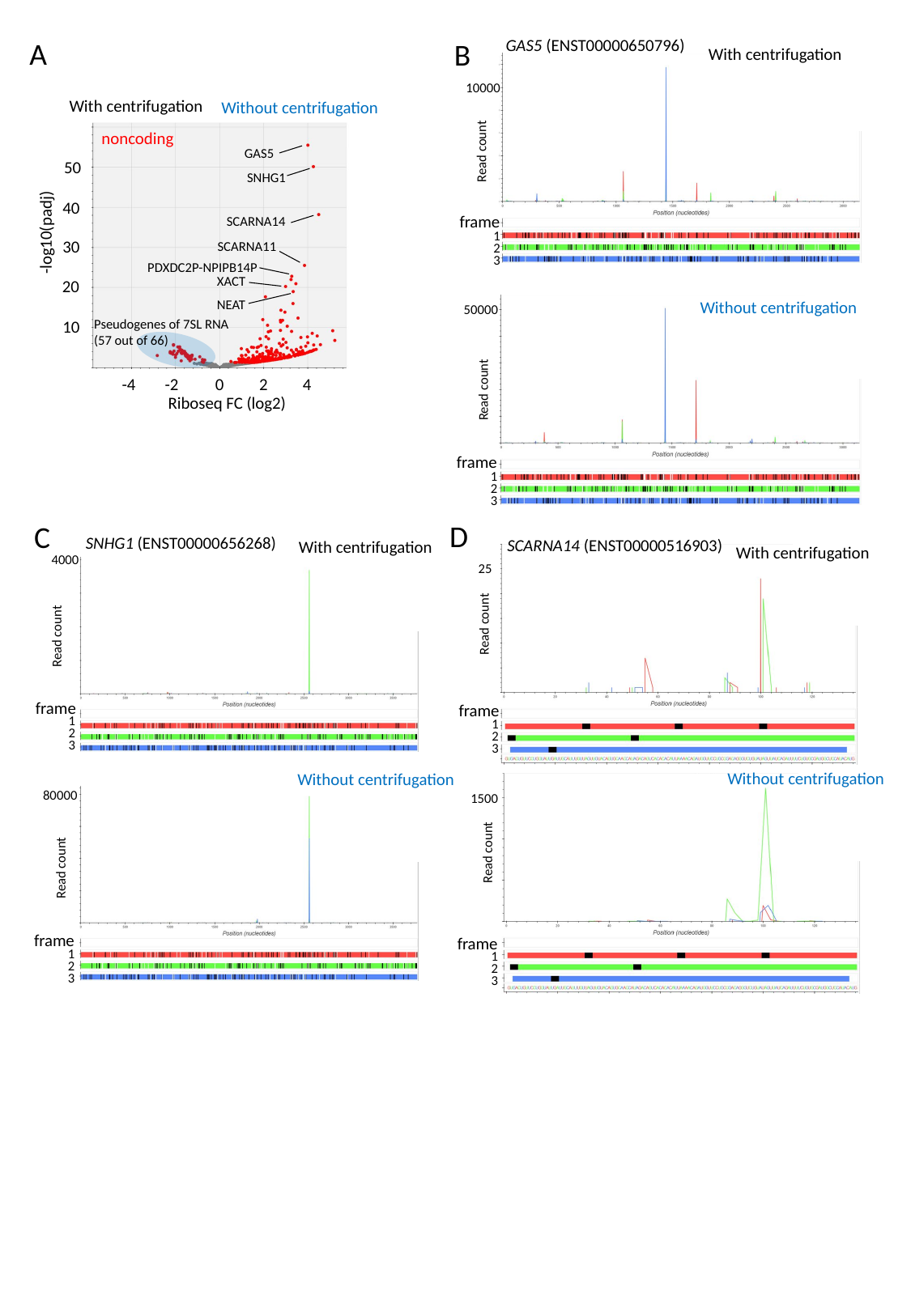

GAS5 (ENST00000650796)
With centrifugation
10000
Read count
frame
1
2
3
Without centrifugation
50000
Read count
frame
1
2
3
A
With centrifugation
Without centrifugation
noncoding
GAS5
50
SNHG1
40
SCARNA14
-log10(padj)
30
SCARNA11
PDXDC2P-NPIPB14P
XACT
20
NEAT
Pseudogenes of 7SL RNA
(57 out of 66)
10
-4
-2
0
2
4
Riboseq FC (log2)
B
D
SCARNA14 (ENST00000516903)
With centrifugation
25
Read count
frame
1
2
3
Without centrifugation
1500
Read count
frame
1
2
3
C
SNHG1 (ENST00000656268)
With centrifugation
4000
Read count
frame
1
2
3
80000
Read count
frame
1
2
3
Without centrifugation
