# Supplementary material for: Common cell lysis procedures distort ribosome profiling analyses of gene expression
Source: Genome Biol. 2025 Aug 11;26:241. doi: 10.1186/s13059-025-03651-1 (PMC12341276; doi:10.1186/s13059-025-03651-1)
Supplement: Supplementary file 9 — Additional file 9: Fig. S3. Analysis of the effects of omission of centrifugation on riboseq and RNAseq reads aligned to MACF1 genomic locus A) Genomic alignments of riboseq with centrifugation (blue) and without centrifugation (red), related to Fig 2B, and alignments of RNAseq from cell supernatant (blue) and cell pellet (red), related to Fig. 2B, and alignments of RNAseq from cell supernatantand cell pellet, related to Fig. 2A. Region where read density increases in centrifugated and supernatant samples is highlighted in blue. First exon of shorter mRNA isoform which likely corresponds to NM_001397473.1 contain multiple translated uORFs which are highlighted by violet arrows. B) RNAseq reads aligned to longer mRNA ENST00000361689 of supernatant and pellet samples related to Fig. 2A. [file 13059_2025_3651_MOESM9_ESM.pptx]

## Slide 1
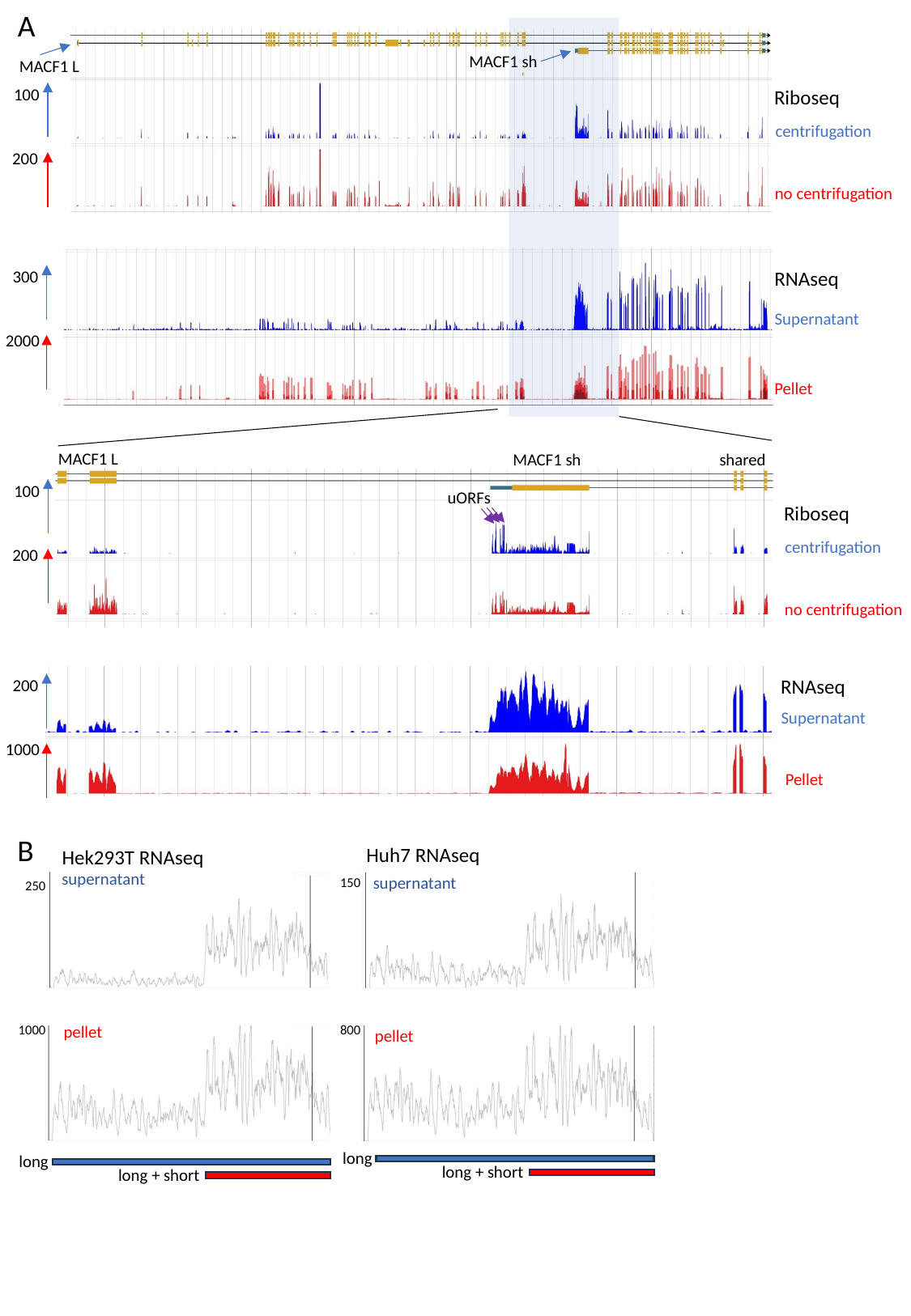

A
MACF1 sh
MACF1 L
100
Riboseq
centrifugation
200
no centrifugation
RNAseq
300
Supernatant
2000
Pellet
MACF1 L
MACF1 sh
shared
100
uORFs
Riboseq
centrifugation
200
no centrifugation
RNAseq
200
Supernatant
1000
Pellet
B
Huh7 RNAseq
Hek293T RNAseq
supernatant
supernatant
pellet
pellet
long
long
long + short
long + short
150
250
800
1000
